# Disease ontologies for knowledge graphs

**DOI:** 10.1186/s12859-021-04173-w

**Published:** 2021-07-21

**Authors:** Natalja Kurbatova, Rowan Swiers

**Affiliations:** 1 Data Infrastructure & Tools, Data Science & Artificial Intelligence, R&D, AstraZeneca, Cambridge, UK; 2 Quantitative Biology, BioPharmaceuticals R&D, AstraZeneca, Cambridge, UK

**Keywords:** Ontologies, Knowledge graph, Data integration

## Abstract

**Background:**

Data integration to build a biomedical knowledge graph is a challenging task. There are multiple disease ontologies used in data sources and publications, each having its hierarchy. A common task is to map between ontologies, find disease clusters and finally build a representation of the chosen disease area. There is a shortage of published resources and tools to facilitate interactive, efficient and flexible cross-referencing and analysis of multiple disease ontologies commonly found in data sources and research.

**Results:**

Our results are represented as a knowledge graph solution that uses disease ontology cross-references and facilitates switching between ontology hierarchies for data integration and other tasks.

**Conclusions:**

Grakn core with pre-installed “Disease ontologies for knowledge graphs” facilitates the biomedical knowledge graph build and provides an elegant solution for the multiple disease ontologies problem.

## Background

Disease ontologies are used for annotation, integration and analysis of biological data, and knowledge graph construction. The range and diversity of disease ontologies are high due to various specific areas they are used in, e.g. medical practice, rare disease domain, biological experiments and biobanks. To build a biomedical knowledge graph, we integrate data from different databases that may use different disease ontologies and from publications where authors also have their preferred ontologies. The following sentence can describe the data integration challenge: one disease—multiple disease terms (IDs) and hierarchies originating from different disease ontologies.

There are two parts to the challenge: firstly, we require matches between different disease ontologies; secondly, we need a system that can exploit this matching, e.g. perform data queries that can collect data from the desired disease hierarchy, in order to map one ontology to another. Ontological matching is a separate research area with a number of findings and approaches [1–3]. We used cross-reference information (ontological matching results) in disease ontologies to collect matchings and curation to achieve the atomicity of the mappings needed for this project. We used Grakn logical reasoning to solve the second challenge: switching between different ontologies and their hierarchies to integrate data and retrieve a particular disease domain view onto the disease of interest.

There are two conventional approaches to data integration: “data factory”, where the data is integrated before ingestion into the knowledge graph and data integration on the fly, where the data is integrated directly inside the knowledge graph. We used a combined approach in this work—disease ontology data is pre-prepared using R scripts before loading. Simultaneously, we used a database schema that supports ad-hoc data integration, leading to flexible data loading and reasoning. We are not changing the disease ontology data per se or factoring the data to use one specific ontology; rather, we combine existing information and focus on exact matching terms, leaving the data integration task to the database. The keyword here is flexibility: a user can easily change data prepared for loading, focusing on a disease area of interest and adding more ontologies, including custom ones.

## Implementation

### Data preparation

We created a matching file using R scripts to extract cross-referencing data from ontologies of interest.

**Fig. 1:**
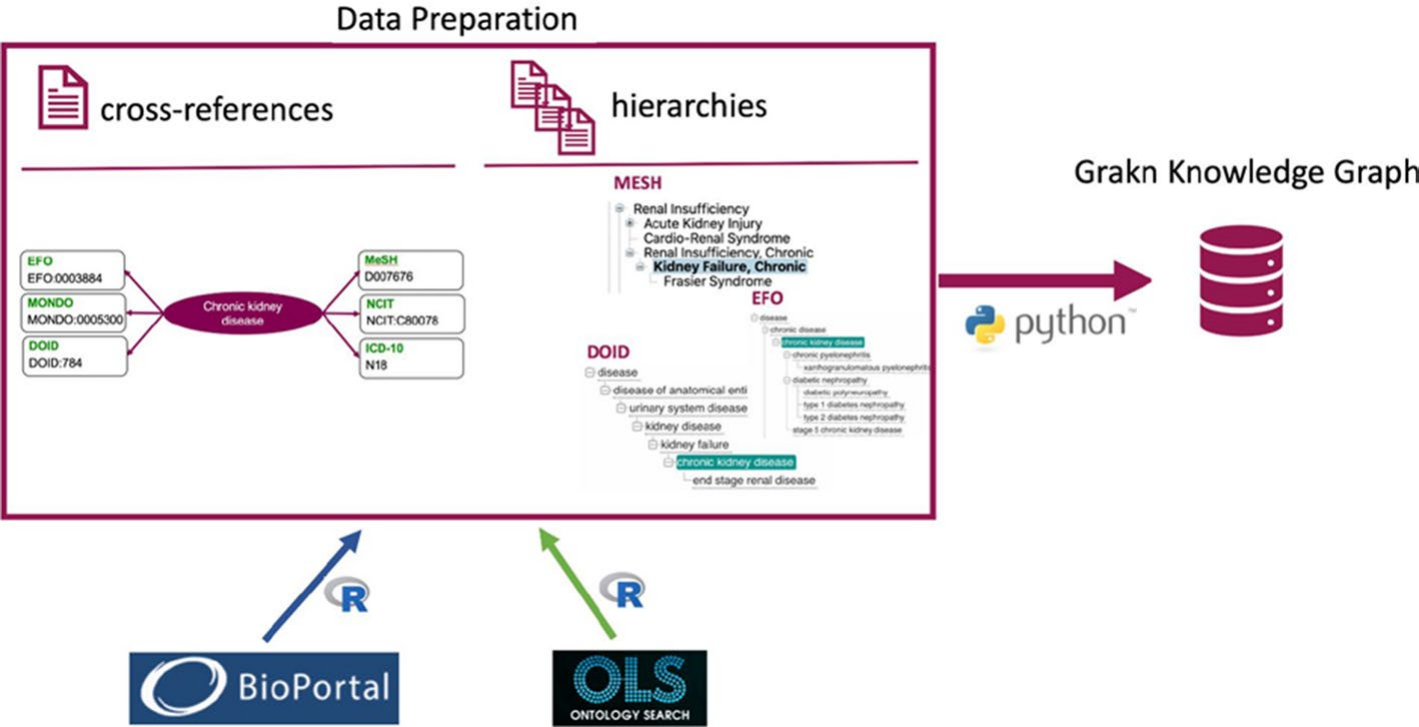
The disease ontology basis for the knowledge graph. Data Preparation: ontology matching presented as cross-reference flat-file and ontological hierarchies are created using Bioportal and Ontology Lookup Service data processed by R scripts. Chronic kidney disease and its presentation from six disease ontologies perspective are shown as a diagram to give an example of a cross-reference file record. Grakn Knowledge Base: data is loaded into the database from data files with python scripts

There are 21,696 records in the matching file (./data/prepared_ontologies/cross-reference.tsv). We used Bioportal [4] and Ontology Lookup Service [5] to collect up-to-date cross-reference information from the following ontologies: MeSH [6], UMLS [7], EFO [8], NCIT [9], OMIM [10, 11], DOID [12], Orphanet [13], HP [14], MONDO [15] and ICD-10 [16]. These particular disease ontologies were chosen pragmatically—EFO, Orphanet, DOID, HP, NCIT, OMIM and MONDO are broadly used in biomedical databases and archives. MeSH is used for indexing articles in PubMed [17] and as a result, is the primary source of disease referencing in document retrieval systems and Natural Language Processing (NLP) pipelines [18–20]. UMLS was included as a single source of cross-referencing for some of the disease ontologies. We added ICD-10 for genomic data integration from UK Biobank [21]. To build the foundation for biomedical data integration, we are interested in atomic matching between disease ontology terms. Formally, we define ontological matching as a triple $m= <{t}_{id}, {t}_{j}, s>, s\in \{\mathrm{0,1}\}$, where ${t}_{id}$ is the preferred disease term from the ontology that defines the disease label, s is the binary similarity degree. An atomic mapping in this matching is a pair $\mu = <{t}_{id}, {t}_{j}>$, where ${t}_{id}$ and ${t}_{j}$ are homogeneous ontology terms from the list of ontologies mentioned above. For example, the record from cross referencing file for “chronic kidney disease” (Fig. 1) shows that the disease term has ${t}_{id}$ = “MONDO_0005300” and defines 6 matching pairs: $\mu = <{t}_{id}, {t}_{j}>$, where ${t}_{j}\in$ {MeSH:“D007676”, UMLS:”C0022661”, EFO: “EFO_0003884”, NCIT:”NCIT_C80078”, DOID: “DOID_784”, ICD-10: “N18.9”}. This induces 6 triples of the form $<{t}_{id}, {t}_{j}, 1>$ in our ontological matching, all other ${t}_{j}$ will map to. The MONDO ontology is chosen to represent preferred terms since it covers most of the terms from other disease ontologies. However, the preferred ontology can be changed by user preference. We chose to only consider exact matching terms rather than close matches to reduce noise and prevent problems in the ontology merging. We do not lose too much information as several Ontologies have more exact matches than close matches.

In the last few years, ontological matching quality and amount of cross-referencing data present in disease ontologies has improved significantly. However, there are references to obsolete terms, absence of matching, one source for ontological matching (UMLS in the case of NCIT), ontological matching to parental terms instead of atomic matching (a complex type of matching) and other issues. By combining multiple ontologies and their cross-referencing information, we validated cross-references, found discrepancies and/or matchings that are not atomic and fixed them. There are two types of discrepancies: reference to non-existing term (ontology A references ontology B where the referenced term is obsolete); reference to all hierarchical levels (ontology A term a references ontology B terms b, b_1_, b_2_, …, b_n_ where b_1_, …, b_n_ are children of b). In the latter case, nothing is incorrect from an ontology A perspective. However, it is not an atomic reference, and for our purpose of atomic matching, we had to fix this type of reference (ontology A term a is referenced to ontology B term b).

Changes were done only on the level of the cross-reference file that is available on Github repository. The user of the software can change cross-references if needed. The only principle that should be held in place for the intended functionality is the atomicity of the matchings.

We believe that disease cross-references in a flat file that is easily accessible and editable will improve ontological matching in particular disease areas. Disease ontology hierarchies is another source of data for the project. We use ontologies from Bioportal and R scripts to extract relevant hierarchical information based on the matching file described above. The GitHub repository explains how to repeat the data preparation process. Table 1 describes in detail individual ontology contributions into cross-referencing and unique terms.

**Table 1:** Individual ontology contribution into cross-referencing and unique terms

Ontology/counts	# Terms only in this ontology	# Preferred terms	# References	# Unique references
MESH	0	0	8328	8251
UMLS	0	0	17,648	17,591
EFO	7	70	4930	4930
NCIT	0	24	7067	7067
OMIM	0	0	8056	8032
DOID	0	5	9001	9001
Orphanet	1	69	9066	9066
HP	80	75	652	652
MONDO	109	21,453	21,482	21,482
ICD10	0	0	11,271	4103
Total	1186	21,696	97,501	

Column "Number of terms only in this ontology" shows the number of unique terms from the ontology (when there are no cross-references in other ontologies); column "number of preferred terms" presents the number of terms that were used as the main entries (while other ontologies provided cross-referencing terms), column "number of references" sums up a number of unique terms and cross-references found in the ontology, the last column "number of unique references" shows the number of not repeated references

### Grakn knowledge base

**Fig. 2:**
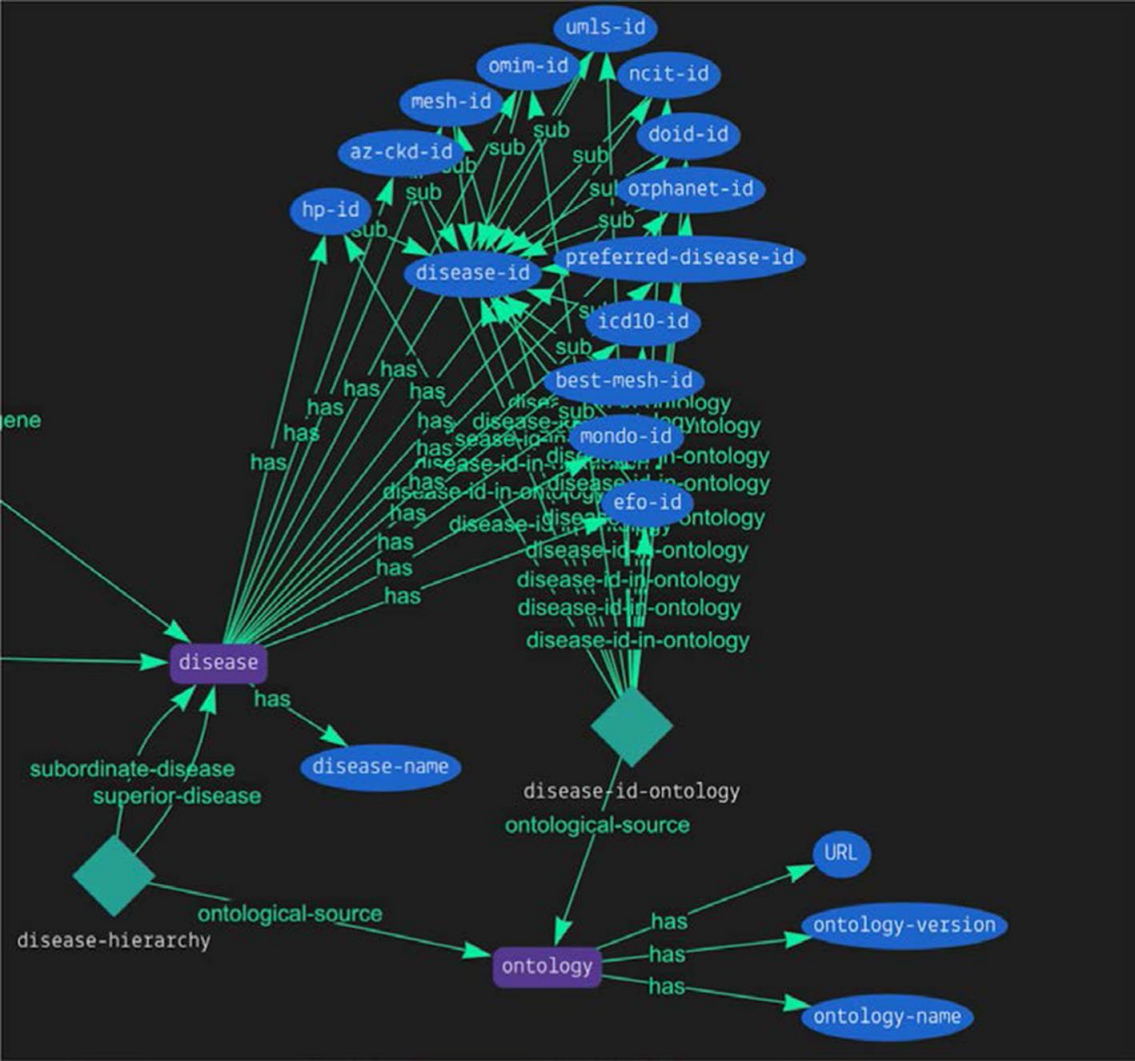
Grakn Knowledge Base: part of the schema diagram shows a disease node with multiple attributes for ontological terms. Grakn schema with all nodes, attributes and logical rules is available at Github repository

We provide a Grakn schema with logical rules to make ontological inferences and a preloaded Grakn database. Example queries and use cases are available together with loading scripts written in python to rebuild and extend the database. Figure 2 shows a schema diagram for a disease node with multiple attributes for ontological terms. The Grakn database was chosen due to its flexible schema and its logical reasoning capabilities, allowing us to switch between different disease ontologies with ease or to incorporate all available ontological hierarchies together for an overall view of a particular disease. Grakn’s logical reasoning engine supports transitivity rules essential for ontological matching [22]. From a practical perspective, transitivity rules enable access to all the children of a particular disease term in a straightforward query and the use of multiple disease ontology hierarchies together, e.g. to get all subordinate diseases for a particular disease considering all available ontologies.

## Results

Our results consist of a Grakn knowledge base, schema and loading scripts that allow the building of a biomedical knowledge graph foundation—creating a practical solution that allows easier data integration from NLP pipelines and a variety of biomedical databases. This knowledge graph solution enables comprehensive exploration and interaction with disease ontologies. It visualises disease ontologies, allowing query of all sub-classes of a particular term regardless of the ontology using one command, facilitating switching between different ontologies, and remapping one ontology terms, e.g. MeSH, to the hierarchical structure of another ontology (e.g. MONDO).

After loading over prepared data, obtaining all sub-classes of "chronic kidney disease" from the available hierarchies of disease ontologies is now trivial using Graql query:
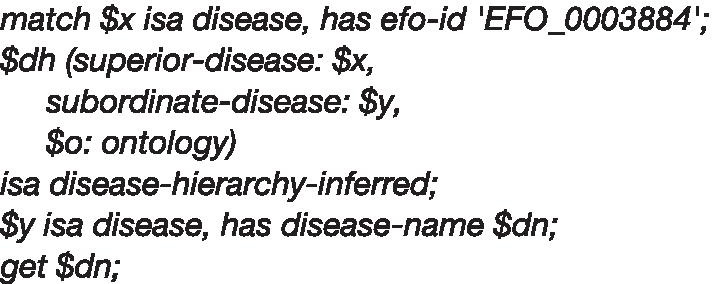


Multiple other common ontological problems are solved, and examples are available in the Github repository.

We also provide data preparation R scripts to process disease ontology data in a format understandable for the Grakn knowledge base, together with pre-processed data files for MONDO, DOID, EFO, HP, MESH, Orphanet, UMLS, ICD-10 and NCIT disease ontologies.

## Conclusions

Disease ontologies for knowledge graphs is a knowledge base solution that uses Grakn core with its logical inference and disease ontologies cross-references to allow easy switching between ontology hierarchies for data integration purpose. This software makes it straightforward to run common ontological queries. It is relatively easy to add new ontologies due to the python loading scripts, and the Grakn reasoning rules are easy to extend. We hope this software will make it easier for bioinformaticians to integrate data that uses multiple ontologies.

## Availability and requirements

Project name: Disease_ontologies_for_knowledge_graphs; Project home page: https://github.com/natacourby/Disease_ontologies_for_knowledge_graphs; Operating system(s): Platform independent; Programming language: Python, R; Other requirements: The community edition of Grakn Core License: Affero GPL v3; Any restrictions to use by non-academics: no.

## Data Availability

The datasets generated and/or analysed during the current study are available in the Github repository, https://github.com/natacourby/Disease_ontologies_for_knowledge_graphs /tree/master/data.

## Abbreviations

MeSH: Medical subject headings
UMLS: The Unified Medical Language System
EFO: Experimental Factor Ontology
NCIT: NCI Thesaurus
OMIM: Online Mendelian Inheritance in Man
DOID: Human Disease Ontology
Orphanet: Orphanet Rare Disease Ontology
HP: Human Phenotype Ontology
MONDO: Mondo Disease Ontology
ICD-10: International Statistical Classification of Diseases and Related Health Problems: tenth revision
